# Erythropoietic Protoporphyria: You May Not Have Seen It, but It May Have Seen You

**DOI:** 10.7759/cureus.23253

**Published:** 2022-03-17

**Authors:** Larissa M Pastore, Christina W Sun, Sylvia Hsu

**Affiliations:** 1 Dermatology, Cooper Medical School of Rowan University, Camden, USA; 2 Dermatology, Temple University Hospital, Philadelphia, USA

**Keywords:** phototoxicity, cirrhosis, porphyria, photosensitivity, erythropoietic protoporphyria

## Abstract

Erythropoietic protoporphyria is a rare skin condition that commonly presents in childhood. We report a case of a 35-year-old Hispanic male with a history of sun sensitivity, presenting with complaints of immediate burning and itching of the skin on his face and upper extremities upon sun exposure. On examination, there was minimal face erythema and calluses over the knuckles. Laboratory workup demonstrated substantially increased protoporphyrin (over 10 times the upper limit of normal) along with elevated liver enzyme levels. Liver biopsy confirmed stage 4 cirrhosis. Our patient’s cutaneous manifestations were the primary complaint that led to the diagnosis of his terminal hepatic illness. We recommend screening for erythropoietic protoporphyria in patients who present with a life-long history of non-blistering, burning and itching of the skin, which begins immediately upon sun exposure.

## Introduction

Porphyria encompasses a group of rare hereditary hematopoietic diseases that occur due to enzyme abnormalities at various points in the hemoglobin synthesis pathway. Specifically, erythropoietic protoporphyria (EPP) is a form of porphyria caused by a deficiency in the enzyme ferrochelatase (FECH). This enzyme metabolizes protoporphyrins and catalyzes the addition of a ferrous ion into the protoporphyrin ring during the formation of the heme molecule. The prevalence of EPP has been estimated to be 1:75,000 to 1:200,000 [1]. Its onset is usually in early adolescence, and it is the most common form of porphyria in children, seen in roughly 0.5 to 2.7 in 100,000 children [2].

EPP is most commonly an autosomal recessive inherited disease caused by a mutation in the gene encoding FECH located on chromosome 18q21​.31. The result of this enzyme deficiency is a toxic accumulation of protoporphyrins in the bloodstream. The protoporphyrins bind tightly to albumin and are transported to the liver where they accumulate and cause hepatocellular toxicity. Lipophilic plasma protoporphyrins also accumulate in dermal vessels of the skin and are highly sensitive to both ultraviolet and visible light. Once activated, they cause oxygen free radical formation and subsequent injury to the overlying skin [2].

The major presenting symptom of EPP is severe skin pain and photosensitivity upon sunlight exposure. Patients experience tingling, itching, and burning of the skin. After continued exposure to ultraviolet or fluorescent light, the skin becomes markedly erythematous and swollen. The hands, arms, and face are the most affected areas. In contrast to porphyria cutanea tarda, blistering of the skin is absent. Due to the excess protoporphyrins excreted in bile, some individuals may develop complications related to liver and gallbladder dysfunction. Findings may include abdominal pain, jaundice, cirrhosis, hepatomegaly, splenomegaly, and cholelithiasis [2].

## Case presentation

A 35-year-old Hispanic male with a past medical history of hypertension and diabetes mellitus presented with complaints of immediate burning and itching of the skin on his face and upper extremities upon sun exposure. These symptoms began at birth. He also noted episodes of abdominal cramping. The patient recalls that in childhood, he had several episodes of sunburned skin and was often instructed to use sunscreens by his pediatrician. His brother and sister were unaffected, but he reports that his mother also burns easily in the sun.

**Figure 1:**
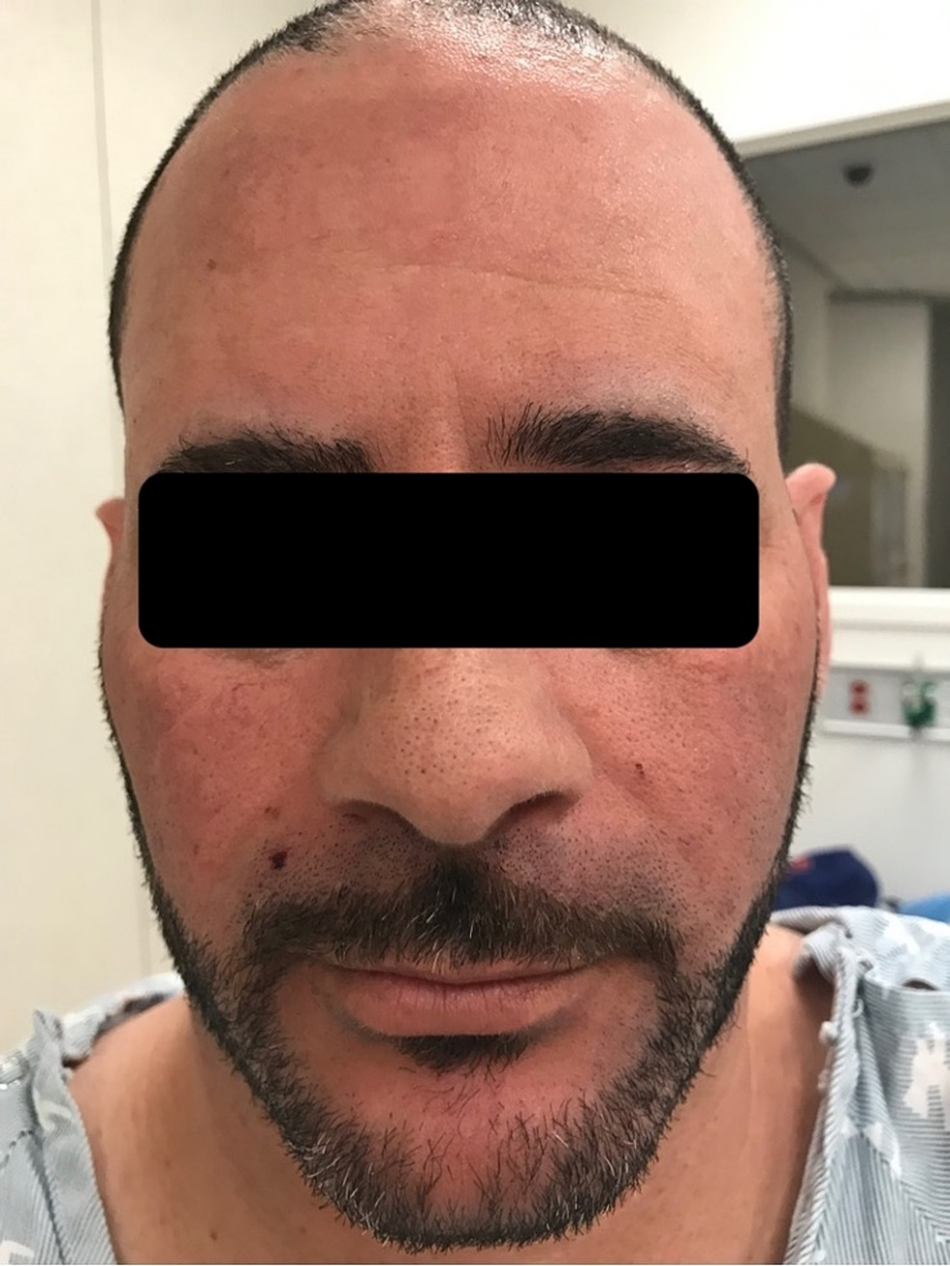
Mild erythema of the face.

**Figure 2:**
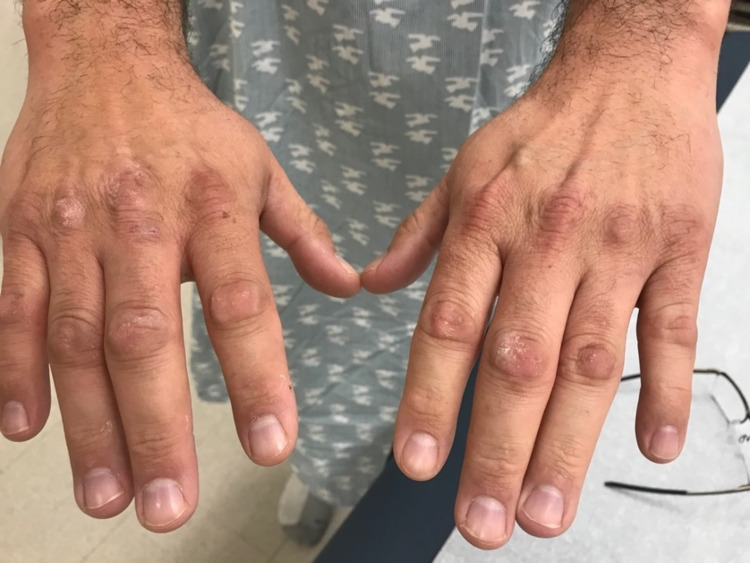
Calluses on the dorsal aspect of the hands.

On physical exam, he had minimal erythema on the face (Figure 1). The dorsal hands revealed calluses over the knuckles, but no blisters or bullae were present (Figure 2). There were no significant cutaneous findings on the lower extremities and mucosal surfaces.

Diagnosis of EPP was confirmed with laboratory studies, which showed elevated total plasma porphyrins at 76.1 mcg/L (normal: 1.0-5.6 mcg/L). Protoporphyrin was elevated at 51.3 mcg/L (normal: 0.4-4.8 mcg/L). The liver enzymes, aspartate aminotransferase (AST) and alanine aminotransferase (ALT), were both elevated at 133 U/L (normal: 10-40 U/L) and 198 U/L (normal: 9-46 U/L), respectively. Complete blood count, albumin, and bilirubin were within normal limits. Alkaline phosphatase was normal at 69 U/L.

Based on the presentation and the abnormally elevated laboratory values, the patient was referred to hepatology and a liver biopsy was recommended. The biopsy revealed focal lobular inflammation composed of lymphocytic infiltrate, microvesicular steatosis, ballooning of hepatocytes, and fibrosis, which were all consistent with nonalcoholic steatohepatitis (NASH). Trichrome stain confirmed stage 4 chronic cirrhosis. Accordingly, the hepatology service recommended periodic screening for hepatocellular carcinoma, utilizing abdominal ultrasound imaging, monitoring of alpha-fetoprotein levels every six months, and maintenance of hepatitis A and B immunity.

## Discussion

The primary treatment for EPP is avoidance of sun exposure and use of sunscreens that are sun protection factor (SPF) 30 or greater. Protection of the skin with garments, such as long-sleeved shirts, trousers, hats, and sunglasses, is also recommended. In addition, our patient was advised to use a medical-grade automobile window tint to prevent severe skin burns. There is no standard medication regimen for the cutaneous manifestations of EPP; however, recent literature suggests afamelanotide, an α-melanocyte-stimulating hormone, helps to reduce photosensitivity and extends pain-free intervals [2,3].

Commonly, symptoms of EPP present inconspicuously in early infancy, but there is often a significant delay in diagnosis. In our patient, there was a history of sunlight hypersensitivity in childhood, but he was diagnosed with EPP only after presenting to our clinic at the age of 35. A study done in Europe showed the median age for diagnosis to be 21 in females and 20.5 in males [4]. Cutaneous manifestations of EPP, such as acute painful skin within minutes after sun exposure and associated edema, always precede hepatic manifestations and tend to occur initially in childhood [3]. The delay in diagnosis is likely because EPP is often mistaken for other skin conditions, such as sunburns, cutaneous allergies, or angioedema. Thus, EPP commonly is overlooked until adulthood when the patient develops acute cutaneous exacerbations, hepatic failure, or incidental findings of elevated liver enzyme levels [5]. Therefore, it is important, even in older patients, to thoroughly assess photosensitivity reactions and obtain appropriate confirmatory blood work, if EPP is suspected.

It has been reported that up to 5% of patients with EPP will go on to develop irreversible liver disease, which necessitates liver transplantation [5]. Although our patient reported abdominal cramping, he had no jaundice, ascites, or encephalopathy, which would have suggested chronic liver failure. The liver biopsy, however, demonstrated features of EPP-induced cirrhosis. If diagnosed early, certain treatments can be initiated, including ursodeoxycholic acid to induce bile flow, parenteral iron and hematin infusions to reduce protoporphyrin production, and N-acetyl cysteine to prevent hepatotoxicity [6]. EPP, which is diagnosed prior to any gastrointestinal symptoms, has been shown to have lower morbidity and better long-term outcome [3]. Therefore, we recommend early screening for EPP in young patients presenting with a chief complaint of non-blistering, painful skin that begins immediately upon sun exposure.

## Conclusions

Our patient presented with a life-long history of burning and painful, non-blistering skin immediately after sun exposure. The diagnosis of EPP was not made until he was 35 years old. By then, the patient had end-stage liver failure, a rare co-morbidity that is associated with EPP. Early recognition of the cutaneous manifestations of EPP will expedite management and lead to better treatment outcomes. It is also important to note that if the practitioner checks a 24-hour urine porphyrin level, the diagnosis of EPP will be missed. The practitioner needs to check either the plasma porphyrins or the stool porphyrins since protoporphyrin is not water-soluble.
